# Reassessing the emergency department burden of influenza: A comprehensive real-world analysis using administrative data

**DOI:** 10.1371/journal.pone.0340699

**Published:** 2026-02-18

**Authors:** Dariush Khezrimotlagh, Sara Imanpour, Erol Akbas

**Affiliations:** 1 School of Science, Engineering, and Technology, Pennsylvania State University, Harrisburg, Pennsylvania, United States of America; 2 School of Business Administration, Pennsylvania State University, Harrisburg, Pennsylvania, United States of America; Taipei Medical University, TAIWAN

## Abstract

Emergency departments (EDs) are burdened by high patient volumes during influenza seasons, yet the clinical and operational profile of influenza visits remains underexamined in large-scale data. This study analyzes a stratified sample of more than 5.5 million patient records from the New York State Emergency Department Database (SEDD) in 2019 to assess the comorbidity burden, outcomes, resource utilization, and readmission patterns of influenza visits compared to all other emergency department encounters. The objective is to determine whether influenza visits reflect higher severity and health system strain or lower-acuity cases suitable for alternative care settings. Influenza visits show lower comorbidity burden, no observed mortality, lower total charges, and shorter lengths of stay. Patients with influenza have fewer overall readmissions, longer average time before return, and substantially lower thirty-day readmission rates than patients with non-influenza diagnoses. These findings indicate that influenza ED visits are lower in complexity and resource burden. By linking comorbidity adjusted severity with real world outcomes, this study provides empirical evidence to guide seasonal care planning and resource allocation.

## 1. Introduction

Emergency department (ED) visits are an essential component of the health care system, providing acute care for individuals with urgent medical conditions. Patterns of ED utilization yield important insights into health seeking behavior and reflect both individual needs and broader systemic factors [1,2]. The U.S. health care landscape has undergone substantial change over recent decades, driven by evolving insurance coverage, advances in medical technology, and policy reforms [3]. These shifts have altered patterns of access and use of health services, including emergency care, particularly in cases that may not require emergency treatment, such as seasonal flu.

Seasonal influenza continues to exert considerable pressure on global health systems, not only due to its contagious nature and morbidity in high risk populations but also because of how it reshapes healthcare demand patterns, especially in E.Ds. Each winter, healthcare facilities experience measurable spikes in visit volume, much of which is attributable to patients presenting with influenza like illness. While the disease burden of influenza has been widely documented in hospitalized populations and through national mortality surveillance, less attention has been paid to the structural and operational strain placed on EDs, which serve as the front lines for unscheduled care [4]. Addressing this gap is crucial as EDs are often overcrowded, and policymakers must prioritize which patient populations can be redirected to more appropriate, less resource intensive settings. Understanding the characteristics of influenza ED visits and how they compare with non-influenza encounters is necessary to inform evidence based resource planning, especially during seasonal surges.

ED utilization during the influenza season imposes significant strain on health system resources, but the clinical necessity of many influenza visits remains unclear. While research has examined inpatient burden [5] and mortality associated comorbidity [6], few studies leverage state ED data to compare influenza versus non-influenza visits across comorbidity, cost, and readmission outcomes. This study analyzes over 5.5 million patient records from the Healthcare Cost and Utilization Project (HCUP) New York State 2019 State Emergency Department Database (SEDD), focusing on ED encounters coded with ICD-10 diagnosis codes J09-J11. These codes represent influenza conditions as defined by the International Classification of Diseases, Tenth Revision (ICD-10). The New York SEDD aggregates records from all non-federal acute care hospitals across the state, covering approximately 150 facilities that submit standardized visit data through HCUP. New York collects ED data from all licensed hospital owned emergency departments in the state [7]. SEDD counts include only ED visits not resulting in an inpatient admission. ED utilization in New York State remained consistently high from 2017 to 2019, with more than 8 million visits annually among 19.6 million residents, representing approximately 41.4 visits per 100 individuals. Both total ED volume and per-capita visit rates declined in 2020, likely reflecting pandemic related shifts in care seeking behavior. Although utilization increased again in 2021, it did not return to pre-pandemic levels. In 2019, approximately 17% of ED visits resulted in an inpatient admission, a proportion that was consistent with prior years. This rate rose to 20% in 2020 and then decreased slightly to 19% in 2021 [7].

Emergency department evaluation relies on established performance metrics that measure flow, timeliness, and resource efficiency. Prior work has shown that ED crowding, long wait times, and variation in throughput are linked to diagnostic complexity and case mix, and these factors influence operational strain during seasonal surges [8,9]. Studies in ED performance analytics use administrative data to assess triage effectiveness, patient flow, and quality in care delivery, providing a framework for interpreting differences in utilization patterns across conditions. These findings show that ED administrative records can support systematic evaluation of patient groups and can help identify conditions that produce lower operational burden. Incorporating this analytical perspective strengthens the motivation for comparing influenza and non influenza visits within a performance evaluation context.

This study quantifies the comorbidity and resource profile of influenza ED visits; evaluates how they compare with non-influenza visits in mortality, cost, length of stay (LOS), and readmission patterns; and assesses whether they offer evidence to support care realignment away from high cost acute care settings. This includes examining whether flu patients differ from others in clinical complexity and trajectory after discharge, indicating whether many of these visits could be managed safely in lower acuity settings.

Previous work has shown that comorbidity adjusted burden influences outcomes in respiratory illness, especially among elderly populations and those with chronic disease [10]. While the role of influenza in hospitalization has been widely studied [11,12], there is limited quantitative evidence regarding its comparative burden in the ED. Literature addressing readmission dynamics for viral illnesses is even more sparse, particularly with respect to ED-to-ED returns rather than hospitalizations [13,14]. Influenza has been observed to drive spikes in ED visits during winter peaks [15,16], but operational decisions on staffing and triage still lack visit level data to support resource reallocation.

Previous literature indicates that influenza places a substantial burden on emergency healthcare services, underscoring the need to incorporate surveillance data into public health planning and influenza management. Among adults aged 18–64, costs increase with age and underlying conditions, with hospitalization expenses reaching up to 2.5 times higher in at-risk populations. Evidence also shows that older adults, particularly those with comorbidities, face a higher risk of complications and increased healthcare utilization. Collectively, these findings highlight the need for continued strategies, beyond current vaccines, to mitigate influenza burden among high risk groups [17–19].

Policy focused frameworks such as the preventable ED use model rely on judgments that can be misleading without robust administrative data [20,21]. Although some studies suggest improved outcomes through urgent care or telemedicine for low acuity cases [21,22], ED based triage patterns for influenza remain underexplored. Our work seeks to fill this gap by systematically comparing flu and non-flu ED visits using a large administrative dataset linked to readmission outcomes. This study investigates whether flu ED visits are characterized by lower comorbidity, shorter stays, lower costs, and fewer early returns, which may represent causes that could often be managed outside of the emergency system. This contribution not only reinforces clinical understanding of influenza’s trajectory in acute settings but also provides a quantitative basis for interventions aimed at realigning seasonal care flows.

The rest of this study is organized as follows. Section 2 describes the data and methods; Section 3 presents results; Section 4 discusses implications, and Section 5 concludes the study.

## 2. Data and methodology

### 2.1. Data

To analyze the operational and clinical characteristics of influenza ED encounters, we utilized the 2019 New York SEDD, a comprehensive dataset capturing all outpatient ED visits across the state. The SEDD are created as part of the HCUP, sponsored by the Agency for Healthcare Research and Quality (AHRQ). Each state’s partner data organization, such as a health department, hospital association, or data commission, collects administrative discharge data from non-federal hospitals within that state. These data are extracted primarily from hospitals’ billing and discharge records, not clinical charts. Diagnoses and external causes of injury are coded using the ICD-10 Clinical Modification (ICD-10-CM). Procedures performed in the emergency department are coded using ICD-10-PCS (for hospital reported procedures) or Current Procedural Terminology (CPT) codes, depending on the state’s reporting format.

After cleaning the dataset and missing values, this administrative dataset includes 5,106,148 ED visit records (labeled VisitLinks) with more than 15 million associated diagnosis entries. Our study group consisted of all visits with ICD-10 codes J09-J11 appearing in any of the diagnostic fields, designating them as influenza. All remaining visits were categorized as non-influenza visits. Because the dataset contained over 15 million diagnosis records, it was processed in sequential chunks to accommodate computer’s memory limits during computation and execution of the model.

Key demographic and clinical variables extracted for each encounter included patient age, sex, visit length of stay (LOS), total charges, mortality, Charlson Comorbidity Index (CCI), and three post discharge metrics: a binary indicator for any readmission, the number of days until readmission, and whether the readmission occurred within 30 days. The CCI, widely validated for predicting risk-adjusted outcomes, allowed stratification of patient severity [3,20].

### 2.2. Analytical framework

We first conducted descriptive analyses to summarize differences between influenza and non-influenza ED visits. Central tendency and variability metrics were calculated for each variable within flu and non-flu groups. Comparative metrics included mean CCI, LOS, total charges, and readmission probabilities. We also examined diagnosis and procedure categories using clinical classifications software (CCS) groupings, a tool developed by HCUP that aggregates ICD codes into clinically meaningful categories. The top 10 CCS categories for both flu and non-flu groups were identified based on frequency to characterize the most common visit reasons and allow for focused comparative analysis of utilization patterns across groups.

To quantify the independent association of influenza status with clinical and financial outcomes, we conducted two statistical modeling procedures for binary and continuous outcomes, adjusting for patient age, sex, and comorbidity. A generalized estimating equation (GEE) [23–25] was used to analyze correlated data at the visit level and a generalized linear mixed model (GLMM) [26] was applied to account for individual variability. Bootstrap resampling [27,28] was implemented to assess model stability and derive confidence intervals. Three model specifications were applied. Model 1 estimated the binary outcome of 30-day readmission. Model 2 analyzed total charges as a continuous outcome using CCI as a numerical variable. Model 3 used the same cost outcome but represented CCI categorically to capture potential nonlinear effects.

This framework combines descriptive and multivariable analyses to evaluate differences in severity, cost, and readmission between influenza and non-influenza visits, providing evidence on how influenza affects emergency department utilization during seasonal surges.

## 3. Results and findings

### 3.1. Comparative outcome in the population

Among the 5,106,148 ED visit clusters analyzed in this study, a total of 58,838 were identified as influenza related, comprising 1.15 percent of all encounters. Descriptive comparisons between influenza and non-influenza visits demonstrated a clear divergence in clinical severity and resource use. Visit linkage data showed that most patients had a single ED visit during the study period, confirming that influenza related encounters represent a small share of overall repeat use. As shown in Table 1, Flu visits exhibited a significantly lower average CCI of 0.29, compared to 0.44 among non-flu visits, indicating that flu patients tended to be healthier and less complex. In-hospital mortality was zero for all flu related cases, reinforcing the interpretation that these visits are lower risk. Patients diagnosed with influenza also incurred lower total charges on average ($3,286) than their non-flu counterparts ($3,739), and experienced shorter mean lengths of stay (0.175 vs. 0.202 days).

**Table 1:** Comparison of influenza and non-influenza.

Characteristic	Flu		Non-Flu	
	Mean	Std. Dev.	Mean	Std. Dev.
Age	24.915	21.323	41.885	22.384
CCI	0.290	0.772	0.441	1.035
LOS (days)	0.175	0.481	0.202	0.577
Readmission Count	0.845	0.586	1.411	0.694
Time to return (days)	55.940	72.261	31.341	52.120
30-day readmission rate	0.124	0.060	0.214	0.099
In-hospital mortality	0.000%	0.000%	0.170%	0.081%
Total charges	$3,286.088	$3,969.311	$3,739.068	$5,320.973
Total Count	58,838.000		5,047,310.000	

In terms of care trajectory, flu patients experienced fewer average readmissions than non-flu patients (1.25 vs. 1.97 readmissions), had longer average time to return (55.9 vs. 31.3 days), and had lower 30-day readmission rates (12.4 percent vs. 21.4 percent). These findings collectively suggest that flu related visits are associated with simpler, less costly care episodes that are less likely to lead to near-term ED re-utilization. It is worth noting that analyses performed on sequential data portions (chunks) also confirmed consistent identification of influenza cases and stable outcome patterns across the full dataset.

### 3.2. Diagnosis and procedure category profiling

Diagnosis profiling using ICD-10 categories revealed that flu visits were tightly concentrated around respiratory and viral illness diagnoses. Top categories included “Influenza and pneumonia,” “Acute bronchitis,” and “Other upper respiratory infections,” together accounting for over 70% of flu visit diagnoses. Notably absent from the top ten were cardiovascular, injury, or chronic disease categories, which were common in non-flu visits.

Procedure category summaries showed similar trends. The top CPT categories for flu visits were limited to basic evaluation and management, with little representation of high cost or invasive procedures. This procedural simplicity supports the broader conclusion that flu visits are operationally and clinically distinct from other ED use cases and could be redirected to alternative care settings where appropriate.

### 3.3. GEE bootstrap modeling of readmission

Table 2 summarizes the estimated coefficients and their stability across 1000 resampling using the bootstrap GEE analysis on predictors of 30-day readmission and total charges. In Model 1, influenza status had a negative coefficient of −0.694 with significant in 100 percent of resamples (all the coefficient’s p-value was below 0.05), showing that influenza visits were associated with a lower probability of readmission than non-influenza visits. Age and comorbidity had small positive effects, while sex was weak and inconsistent. Here sex is a binary variable coded as 1 for female and 0 for male.

**Table 2:** The GEE bootstrap results.

Model	Variable	Mean	Std. Dev.	Prop(p < 0.05)
Model 1: Readmitted30 (Binomial)	AGE	0.002	0.000	1.000
	CCI	0.068	0.008	1.000
	Sex	−0.035	0.014	0.460
	HasFlu[True]	−0.694	0.014	1.000
	Intercept	−1.855	0.018	1.000
Model 2: Total charges (Continuous CCI)	AGE	37.967	0.533	1.000
	CCI	795.221	26.192	1.000
	Sex	129.038	24.403	1.000
	HasFlu[True]	119.565	20.890	1.000
	Intercept	1906.687	27.832	1.000
Model 3: Total charges (Categorized CCI)	AGE	39.006	0.511	1.000
	CCI-Category 1–2	819.884	36.014	1.000
	CCI-Category 3–4	2905.596	144.685	1.000
	CCI-Category 5+	4648.388	253.099	1.000
	Sex	126.218	24.530	0.990
	HasFlu[True]	121.865	20.895	1.000
	Intercept	1891.311	27.839	1.000

Models 2 and 3 used total charges as the dependent variable. Influenza status remained significant in both, with positive coefficients. The CCI entered as a continuous score in Model 2 and as categories in Model 3, produced progressively larger coefficients for higher categories, reflecting higher costs for patients with greater disease burden. Age and Sex were minor contributors relative to CCI.

The values in Table 2 show that influenza status retained a stable and statistically significant association with both outcomes under all model structures, and that CCI remained the most consistent positive predictor of increased utilization and cost.

### 3.4. GLMM bootstrap modeling of readmission

Table 3 summarizes the coefficients from a bootstrapped GLMM applied to 30-day readmission to account for individual variability. Influenza status remained a negative and significant predictor (−0.062 ± 0.001), confirming a lower likelihood of readmission compared with non-influenza visits. Age and CCI were small but consistent positive predictors, while Sex showed a weak and inconsistent effect.

**Table 3:** The GLMM bootstrap results.

Variable	mean	std	Prop(p < 0.05)
AGE	0.000	0.000	0.990
CCI	0.011	0.001	1.000
Sex	−0.004	0.001	0.590
Group Variance	1.000	0.000	0.000
HasFlu[True]	−0.062	0.001	1.000
Intercept	0.142	0.002	1.000

The constant Group Variance (1.00 ± 0.00) shows that the random effect structure was stable across all bootstrap iterations, indicating no change in variability among individual patients. The intercept (0.142 ± 0.002) remained highly significant, confirming that the model’s baseline prediction of readmission probability was consistent across resamples.

## 4. Discussion

Our findings are novel and offer new insights into strategies for managing influenza cases to alleviate overcapacity in emergency department settings. These results align with prior research emphasizing the importance of redirecting non-urgent influenza cases to alternative care settings, such as primary care clinics or urgent care centers [29,30]. Encouraging influenza vaccination, particularly among individuals at higher risk for infection, may help reduce overall influenza incidence and, consequently, decrease emergency department visits related to influenza.

The low-acuity profile identified for influenza encounters is also consistent with performance evaluations that use ED metrics to characterize operational burden [8,9]. Within these frameworks, conditions associated with limited diagnostic complexity and minimal procedure use contribute relatively little to crowding pressure, even when they represent a large share of visit volume. The empirical patterns in this study show that influenza follows this profile, indicating that its seasonal surge affects volume more than resource strain. This distinction helps clarify why alternative care pathways for influenza can reduce congestion without compromising clinical safety.

The findings indicate operational opportunities to improve emergency department efficiency during influenza seasons. Targeted diversion strategies include enhanced patient assessment protocols, community influenza clinics, urgent care centers, and telemedicine platforms to improve ED efficiency and reserve resources for higher acuity cases. Integration of real time surveillance and predictive analytics can further optimize seasonal staffing, supply allocation, and care routing during periods of heightened respiratory illness prevalence.

## 5. Conclusion

This study provides a comprehensive, real-world evaluation of influenza ED encounters using over 5.5 million administrative records from New York State. Our analysis shows that influenza ED visits are associated with lower comorbidity, reduced charges, shorter stays, and lower 30-day readmission than non-influenza visits, with no hospital mortality. These results were consistent across different randomized selections of data, variable definitions, and model structures, confirming stability across the dataset.

Several limitations should be noted. The analysis used retrospective administrative data, which describe associations but not causation. Influenza and comorbidity identification relied on ICD-10 coding, which may include miscoding or underreporting. The dataset did not include laboratory data, vaccination status, or viral subtype, and results apply only to one U.S. state and year.

Within these boundaries, the findings show that influenza visits constitute a lower-acuity portion of emergency department activity and provide quantitative evidence that can support seasonal planning and resource allocation. The framework combining descriptive comparisons and bootstrapped multivariable models demonstrates the usefulness of administrative data for large-scale health-services evaluation. Future analyses including broader geographic data and clinical variables could extend these observations and evaluate their persistence across different influenza seasons.
